# A Case of Pigmented Villonodular Synovitis

**DOI:** 10.7759/cureus.25957

**Published:** 2022-06-15

**Authors:** Akhil Sugandhi, Sai Kanth Sharma Kondaveeti, Ashok Sunder

**Affiliations:** 1 Internal Medicine, Tata Main Hospital, Jamshedpur, IND

**Keywords:** arthroscopic synovectomy, monoarthritis, synovial proliferation, pigmented villonodular synovitis, tenosynovial giant cell tumor

## Abstract

Tenosynovial giant cell tumors (TGCT) are a rare group of generally non-malignant tumors that involves the synovium and tendon sheath. A young female patient presented to the outpatient department with a complaint of unilateral knee swelling and pain. She was evaluated as such and based on a provisional diagnosis of benign synovial proliferation, she was treated with an arthroscopic resection. We discuss our case and discuss the possible medical therapies to prevent recurrence.

## Introduction

Tenosynovial giant cell tumors (TGCT) are a rare group of typically non-malignant tumors that involve the synovium and tendon sheath. Localized TGCT is sometimes referred to as giant cell tumor of the tendon sheath and diffuse TGCT is also called pigmented villonodular synovitis (PVS) [1]. PVS is more aggressive and associated with a higher recurrence rate than localized TGCT which necessitates a closer follow-up and possibly use of adjuvant medical therapy to prevent recurrence [2,3].

## Case presentation

A 32-year-old female patient presented to us with a three-month history of pain and swelling over the left knee. The symptoms were insidious in onset and had progressed gradually. At the time of the presentation, the pain was severe, limiting the patient's daily activities. Examination revealed a swollen left knee with limited range of movement owing to the pain.

**Figure 1:**
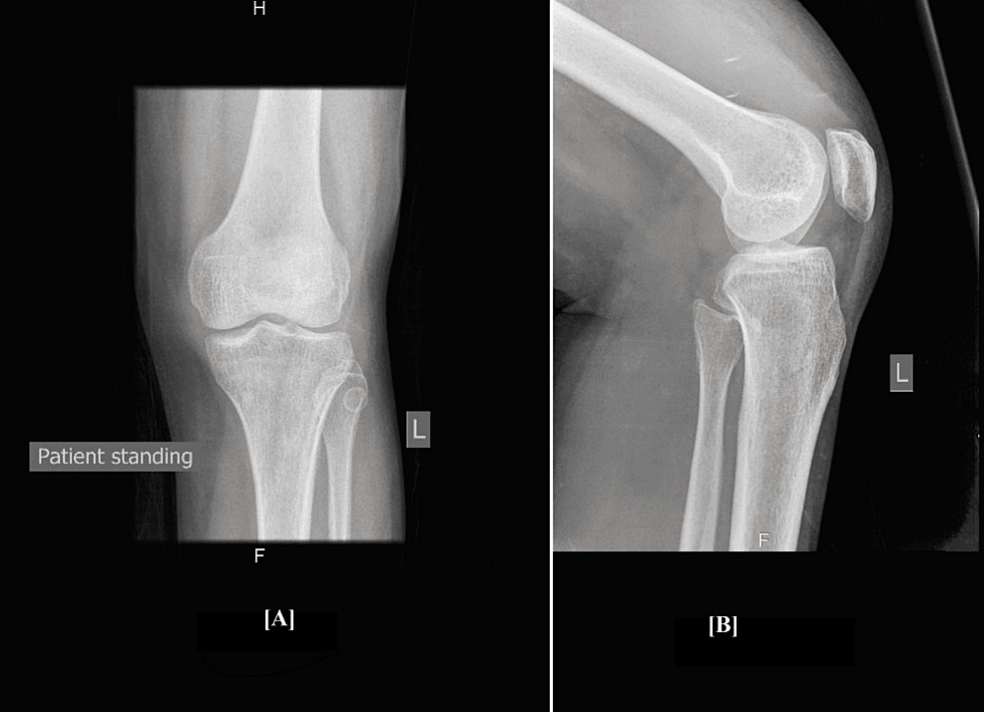
AP view (A) and lateral view (B) x-ray of the knee. The x-ray was unremarkable. AP: anteroposterior

The patient did not have a history of trauma, fever, chills, or rigors. She had been to other hospitals prior to presenting to us. Uric acid levels were normal and rheumatoid factor, anti-nuclear antibody, anti-double stranded DNA antibody, and anti-cyclic citrullinated peptide antibody were all negative. She had been on a trial of antibiotics and analgesics. An x-ray of the knee joint was done and was unremarkable (Figure 1).

**Figure 2:**
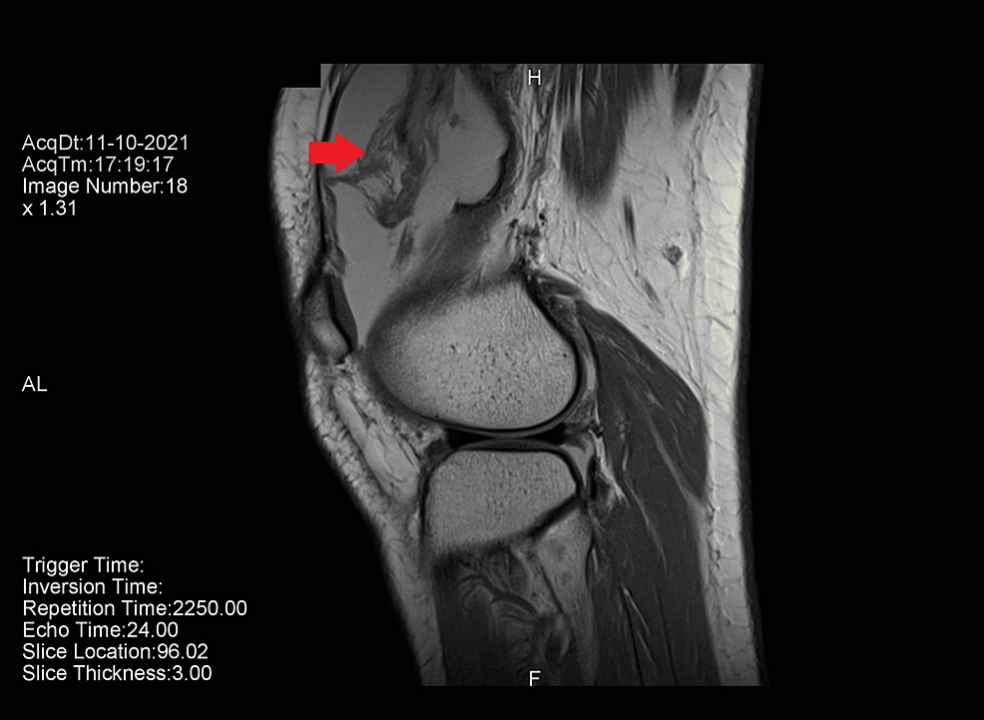
MRI of the knee joint in a longitudinal section (arrow points to the tumor). The tumor is surrounded by an effusion.

**Figure 3:**
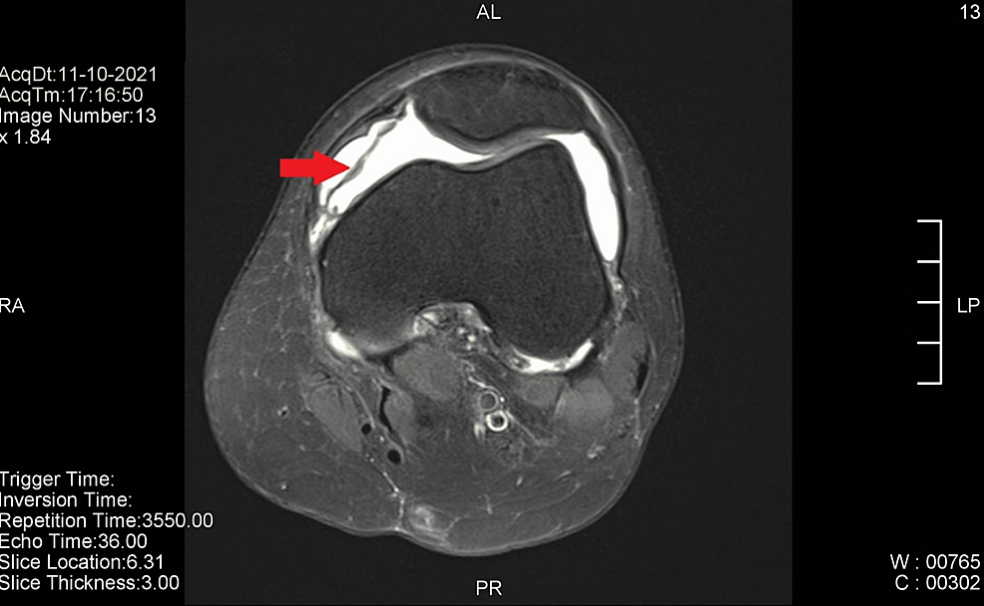
MRI of the knee joint (cross-sectional view). The red arrow points to the tumor.

At this point, an MRI of the knee joint was done, revealing diffuse synovitis with huge reactive knee effusion with extension to both the parapatellar and suprapatellar spaces (Figures 2, 3). The patient was referred to the orthopedics team. An arthroscopic synovectomy was done and sent for histopathological examination.

**Figure 4:**
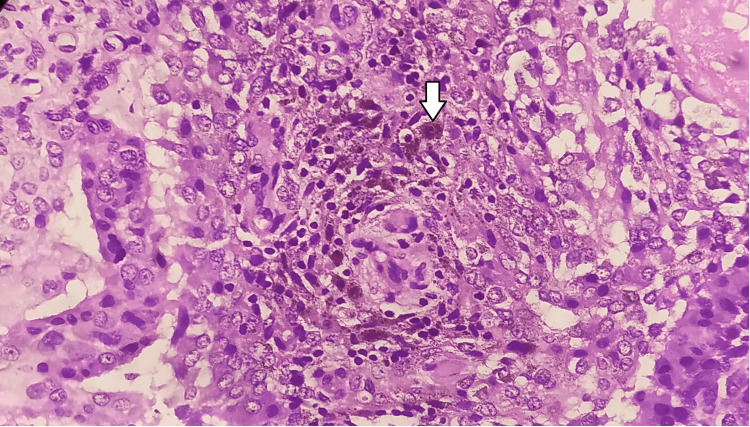
Histopathological slide showing hemosiderin-laden histiocytes (arrow points to a hemosiderin-laden histiocyte).

The synovial fluid revealed a cell count of 50/mcl, all of which were lymphocytes. Culture yielded *Escherichia coli* resistant to multiple drugs, possibly a contaminant in view of the lack of a neutrophilic effusion and multidrug resistance. The biopsy revealed subsynovial nodules composed of stromal cells, osteoclast giant cells, and pigment (hemosiderin) laden histiocytes which are responsible for the reddish brown pigmentation (Figure 4).

Congested and dilated blood vessels with thick hyalinised fibrocollagenous tissue were also seen. This pointed toward the diagnosis of diffuse pigmented villonodular synovitis. The patient was discharged post synovectomy. She was stable and pain-free.

## Discussion

Pigmented villonodular synovitis is a usually benign proliferation of the synovium [1]. It is typically monoarticular, the knee joint is the most common joint involved. It is also known to affect the hip, ankle, shoulder, and elbow in that order of frequency [2]. It is a rare condition affecting about two individuals per million per year. Individuals are usually affected in the second to fifth decades of life [2-4]. The localized form is twice as common in females and the diffuse form has a slight female predominance [1,2]. Our patient's presentation was typical in terms of her age and sex.

The term was coined by Jaffe et al. in 1941 to describe a lesion arising out of the synovium [4,5]. In 1967, Granowitz and Mankin described two subtypes of the conditions, the limited type and the diffuse type [6].

Initially thought to be inflammatory, later evidence suggested a neoplastic origin for the condition. A study by Sciot et al. revealed monoclonality in 58% of the patients with localized and 75% of the patients with the diffuse type [2,3,7]. Genetic data indicate that 1p11-13 is the region most frequently involved in structural rearrangements.

Diagnosis is based on a combination of clinical, radiological, and histological findings. X-rays may show degenerative changes or maybe normal as seen in our case. MRI can demonstrate hemosiderin deposits and help in establishing the diagnosis. Histology remains the gold standard [1,2,8]. Our patient was treated with an arthroscopic synovectomy.

Traditionally the treatment has been excision of the tumor, with debate centered on an open versus arthroscopic approach. Considering the neoplastic origin, recent research has focused on chemotherapy and immunological therapies to prevent recurrence [1-3].

PVS has a high rate of recurrence, depending on the approach to synovectomy and the use of adjuvant therapies especially external beam radiotherapy which offers excellent control. Current recommendations vary a lot from center to center. Effective therapy has been evasive owing to the rare incidence and varied presentation of the disease [2,3,9]. Medical therapy is also being investigated in refractory cases including Infliximab and a monoclonal antibody against CSF-1 receptor [9,10].

Our patient is not planned for any preventive therapy because of resource limitations at our hospital. She has been advised to close follow-up.

## Conclusions

Among the many patients that present to the medicine and rheumatology outpatient department, TGCT would be a rare diagnosis. However, it must be suspected in seronegative arthritis with an appropriate clinico-radiological picture. While surgical therapy is curative for the localized type, a more comprehensive approach is required for the diffuse type in view of its high recurrence rate. There is a wide range of approaches to prevent recurrence with varied efficacy. These approaches vary from center to center. A consensus on the best approach has been elusive thus far.
